# TRIM21 aggravates cardiac injury after myocardial infarction by promoting M1 macrophage polarization

**DOI:** 10.3389/fimmu.2022.1053171

**Published:** 2022-11-10

**Authors:** Zhiqiang Li, Xiangdong Liu, Xingxu Zhang, Wenming Zhang, Mengmeng Gong, Xiaoming Qin, Jiachen Luo, Yuan Fang, Baoxin Liu, Yidong Wei

**Affiliations:** Department of Cardiology, Shanghai Tenth People’s Hospital, Tongji University School of Medicine, Shanghai, China

**Keywords:** myocardial infarction, macrophage polarization, inflammation, tripartite motif-containing protein 21, DNA damage

## Abstract

Macrophage polarization followed by myocardial infarction (MI) is essential for wound healing. Tripartite motif-containing protein 21 (TRIM21), a member of E3 ubiquitin ligases, is emerging as a mediator in cardiac injury and heart failure. However, its function in modulating post-MI macrophage polarization remains elusive. Here, we detected that the levels of TRIM21 significantly increased in macrophages of wild-type (WT) mice after MI. In contrast, MI was ameliorated in TRIM21 knockout (TRIM21^-/-^) mice with improved cardiac remodeling, characterized by a marked decrease in mortality, decreased infarct size, and improved cardiac function compared with WT-MI mice. Notably, TRIM21 deficiency impeded the post-MI apoptosis and DNA damage in the hearts of mice. Consistently, the accumulation of M1 phenotype macrophages in the infarcted tissues was significantly reduced with TRIM21 deletion. Mechanistically, the deletion of TRIM21 orchestrated the process of M1 macrophage polarization at least partly *via* a PI3K/Akt signaling pathway. Overall, we identify TRIM21 drives the inflammatory response and cardiac remodeling by stimulating M1 macrophage polarization through a PI3K/Akt signaling pathway post-MI.

## Introduction

Myocardial infarction (MI) is the most common cardiovascular disease, with high morbidity and mortality (1, 2). Revascularization in the acute phase of MI is a primary therapy to reduce cell death. Despite early intervention after MI to improve acute survival rates, the long-term prognosis of these patients remains poor (3). The irreversible cardiomyocyte death triggers an excessive inflammatory response, with the recruitment of immune cells, which can precipitate adverse remodeling and heart failure (HF) (4).

After MI, abundant macrophages are recruited to the injury site and peak at day 3 post-MI (5). These initially infiltrated macrophages exhibit a proinflammatory phenotype (M1 macrophages) that promotes inflammatory response and clears the necrotic debris. Then macrophages polarized into a reparative phenotype (M2 macrophages) that accelerates the extracellular matrix deposition and scar formation (6, 7). Imbalanced macrophage polarization extends inflammatory reaction, resulting in impaired wound healing and adverse ventricular remodeling (8–11). Thus, revealing the underlying mechanism of macrophage polarization after MI allows a better understanding of the pathogenesis of MI and provides novel and improved therapeutic targets.

Tripartite motif-containing protein 21 (TRIM21) is a member of the tripartite motif protein family (TRIMs), which contains E3 ubiquitin ligases (12). Extensive evidence has established the crucial role of TRIM21 in a wide range of biological processes, such as the innate immune response, carcinogenesis, and cell autophagy (13–15). Genetic deficiency of TRIM21 protects mice from transverse aortic constriction (TAC)-induced cardiac oxidant response damage (16). It has also been confirmed that TRIM21 regulates macrophage polarization in bone marrow-derived macrophages (BMDMs) (17). However, the effects of TRIM21 in post-MI macrophage polarization and macrophage-related cardiac repair responses are not fully established.

In the present study, we explored the role of TRIM21 during the post-MI inflammatory response and clarified the molecular mechanisms whereby TRIM21 regulates macrophage polarization *via* the PI3K/Akt pathway.

## Methods and materials

### Data sources

The gene expression data of monocyte-derived macrophages (MDMs) and bone marrow-derived macrophages (BMDMs) treated with lipopolysaccharide (LPS) were obtained from the Expression Omnibus Gene (GEO) website (http://www.ncbi.nlm.nih.gov/geo/), which provides high‐throughput sequencing data freely. The dataset GSE147310 based on platform GPL16791 (18) and GSE158889 based on platform GPL13112 (19) were retrieved and are publicly available. In dataset GSE147310, 10 MDM samples from healthy donors with or without LPS treatment for 18 hours were analyzed (5 unstimulated vs. 5 LPS stimulated). For dataset GSE158889, 6 BMDM samples treated with or without LPS/IFNγ for 12 hours were selected (3 untreated vs. 3 LPS/IFNγ treated). The TRIM family gene expression profile was extracted using the R language for differentially expressed gene (DEGs) identification. The top 25 DEGs in the TRIMs family were visualized in R language. Common and unique upregulated TRIMs gene sets were identified *via* Venn diagrams (http://bioinformatics.psb.ugent.be/webtools/Venn/).

### Mice

C57BL/6J wild-type (WT) mice were purchased from Vital River Laboratory Animal Technology Co. Ltd. (Beijing, China). TRIM21 heterozygote knockout (TRM21^+/-^) mice were generated as described previously (20). TRIM21 HZ mice were crossed with WT mice and then crossed back to obtain homozygous TRIM21 knockout (TRIM21^-/-^) mice. All animal protocols were approved by the Ethics Committee of Shanghai Tenth People’s Hospital of Tongji University School of Medicine (Permit Number: SHDSYY-2020-1734), conforming to the Care and Use of Medical Laboratory Animals Guidelines (Ministry of Health, P. R. China, 1998).

### MI model

The establishment of the MI model was conducted in accordance with a previous description (21). Briefly, weight-matched mice (~8 weeks) were anesthetized by inhaling isoflurane (1-2%) and intubated with tracheal intubation. The skin between the third and fourth intercostal spaces on the left side of the mice was cut, and the muscle and pericardium were separated layer by layer. The left anterior descending artery (LAD) was carefully exposed and ligated with an 8-0 silk thread immediately at the lower edge of the left atrial appendage. After the operation, the chest was sutured with soluble stitches. Sham mice were operated on with the same procedure without ligation of the LAD. Heart tissues were collected at 1,3,5 and 7 days after MI for subsequent experiments.

### Echocardiography

After anesthetizing mice with 1–2% isoflurane, echocardiography was conducted at 5 weeks with Vevo 2100 system (VisualSonics, Toronto, Canada) using an 18-30 MHz transducer. The left ventricle parasternal long axis and mid papillary muscle short axis were recorded for an average of 5 cardiac cycles. The left ventricular ejection fraction (LVEF), fractional shortening (FS), interventricular septal end-diastole (IVSd), interventricular septal end-systole (IVSs), left ventricular internal diameter end diastole (LVIDd) and left ventricular internal diameter end-systole (LVIDs) were measured by a blinded operator.

### Cell culture and adenovirus infection

The murine macrophage cell line RAW264.7 was obtained from ATCC (Manassas, VA, USA) and maintained in Dulbecco’s modified Eagle medium (DMEM) containing 10% fetal bovine serum (FBS) and 1% penicillin/streptomycin at 37°C in a humidified 5% CO_2_ atmosphere. LV-TRIM21 and LV-green fluorescent protein (GFP) were produced by GenePharma (Shanghai, China) and used according to the manufacturer’s introductions. RAW264.7 cells were infected with LV-TRIM21 and LV-GFP at a multiplicity of infection (MOI) of 100 particles per cell for 12 hours. The PI3K inhibitor, LY294002, was purchased from Beyotime (Shanghai, China).

### BMDMs preparation and polarization

BMDMs were isolated from femurs and tibias of female mice (~6 weeks) as previously described (22). DMEM containing 10% FBS (Gibco, Gaithersburg, USA) and 20 ng/mL of macrophage colony-stimulating factor (MCSF, Sino Biological Inc, Beijing, China) was used to differentiate BMDMs. BMDMs were stained with F4/80 (1:200 dilution Abcam, Cambridge, MA, USA) with >90% positive staining to examine the extraction efficiency. Lipopolysaccharide (LPS, 10 ng/mL, Beyotime) was used to stimulate the BMDMs into M1 macrophages at 7 days.

### Primary cardiac fibroblasts isolation

Hearts were isolated from neonatal mice, minced into small pieces, and digested with collagenase II for 4 times 10 minutes each. Cells were collected by centrifugation and strained through a 70 μm filter. After being plated on 6-cm dishes for 1 hour, adherent cells were maintained in DMEM supplemented with 20% FBS in a humidified atmosphere at 37 °C and 5% CO_2_.

### Wound-healing scratch assay

Cardiac fibroblast migration was analyzed using a wound-healing scratch assay. After synchronizing overnight in DMEM containing low serum medium (0.1% FBS), we used a sterile pipette tip to create a linear scratch and cells were treated with different conditioned mediums from RAW264.7 for 24 h. Wound area changes were quantified at 0 and 24 h by Image J.

### RNA-Seq

Total RNA from BMDMs of WT (n=3) and TRIM21^-/-^ (n=3) mice treated with 10 ng/mL LPS for 12 hours was extracted with TRIzol and sequenced with an Illumina HiSeqTM 2500. We analyzed DEGs using the limma R package with absolute log fold change >1.5 and p-value<0.05. The overall distribution and DEGs were visualized with volcano map and heatmap, respectively. Enrichment of biological pathways was conducted by the Kyoto Encyclopedia of Genes and Genomes (KEGG) pathway enrichment analysis.

### Masson trichrome and H&E staining

After 35 days of MI, heart tissues were obtained from 1 mm below the ligation to the apex, cut into sections (5 μm thickness), and embedded with paraffin. The infarcted area was detected with Masson Trichrome with hematoxylin and eosin (H&E) staining kit (Solarbio, Beijing, China) following the manufacturer’s instructions. The images were recorded with a microscope (Olympus, Tokyo, Japan), and the infarcted size was calculated with ImageJ software.

### Immunofluorescence staining

Heart tissues and macrophages were fixed and permeabilized with 4% paraformaldehyde containing 0.1% Triton X-100 for 10 min at 4°C. After blocking with 5% bovine serum albumin (BSA) for 30 min, the slides were then incubated with primary antibodies against TRIM21 (1:100 dilution, Santa Cruz, CA, USA), F4/80 (1:200 dilution Abcam), γ-H2AX (1:200 dilution, Abcam), iNOS (1:200 dilution, Cell Signaling Technology, CST, Danvers, MA, USA), and Arg1 (Proteintech Group, Chicago, IL, USA) at 4°C overnight and Alexa Fluor 488/594-conjugated secondary antibodies for 1 hour at room temperature. Apoptosis cells were stained with a terminal deoxynucleotidyl transferase-mediated dUTP nick end labeling (TUNEL) apoptosis detection kit (Roche, Mannheim, Germany). Nuclei were stained with DAPI. Images were obtained with fluorescence microscopy, and positive cells and the relative mean fluorescent intensity of six random fields were analyzed with Image J.

### RNA isolation and quantitative real-time polymerase chain reaction

Total RNA from the heart tissue homogenates and cells was extracted with TRIzol reagent (Invitrogen, Carlsbad, CA), and reverse transcription was performed with the PrimeScript RT Reagent Kit (Takara Biotechnology, Dalian, China). Quantitative real-time polymerase chain reaction (qRT-PCR) was completed with Hieff^®^ qPCR SYBR Green Master Mix (No Rox) (Yeasen, Shanghai, China) in a LightCycler 480 Real-time PCR System (Roche) according to the manufacturer’s introduction. Primer sequences used in the study are shown in **Supplement Table 1**. All calculations of relative target gene expression were conducted by the 2^-ΔΔCt^ method with normalization to GAPDH expression (23).

### Western blot

Proteins from LV tissues and cells were extracted in RIPA buffer and quantified with a BCA protein assay kit. Then, the extracts were separated with SDS-PAGE and transferred to a PVDF membrane (Millipore, Billerica, MA, USA). Next, the membrane was blocked with 5% nonfat milk containing 0.1% PBST for 1 hour at room temperature and subsequently incubated with primary antibodies overnight at 4°C. After washing with PBST and incubating with the appropriate secondary antibody, the bands were visualized using ECL Western Blotting Detection Reagent (Tanon, Shanghai, China). Primary antibodies used in the study are listed as follows: TRIM21 (Abcam), F4/80 (Abcam), Bcl-2 (CST), Bax (CST), γ-H2AX (Abcam), iNOS (CST), and Arg1 (Proteintech Group, Chicago, IL, USA) phosphatidylinositol 3 kinase (PI3K, CST), p-PI3K (CST), protein kinase B (AKT, CST), p-Akt (CST), β-actin (CST), Vinculin (Santa Cruz) and glyceraldehyde-3-phosphate-dehydrogenase (GAPDH, CST).

### Statistical analysis

Data are presented as means ± standard deviations. Data normality was determined by the Shapiro-Wilk test. Analysis between two groups was conducted with two-tailed Student’s t-tests, whereas data from three or more groups were compared using one-way ANOVA followed by the Bonferroni *post hoc* test. Nonparametric data were analyzed with the Mann-Whitney U test from 2 groups or the Kruskal-Wallis test with the Dunn *post hoc* test from multiple groups. Kaplan–Meier method and log-rank test were applied to analyze the survival rate changes. All analyses were performed with GraphPad Prism 7.0 (Graph Pad Prism Software, Inc, San Diego, CA) and SPSS 20.0 (SPSS Inc., Chicago, IL, USA). A two-sided P value<0.05 was considered statistically significant. (*P<0.05, **P<0.01, and ***P<0.001).

## Results

### TRIM21 expression is highly increased in the infarct area after MI and in macrophages exposed to LPS

**Figure 1:**
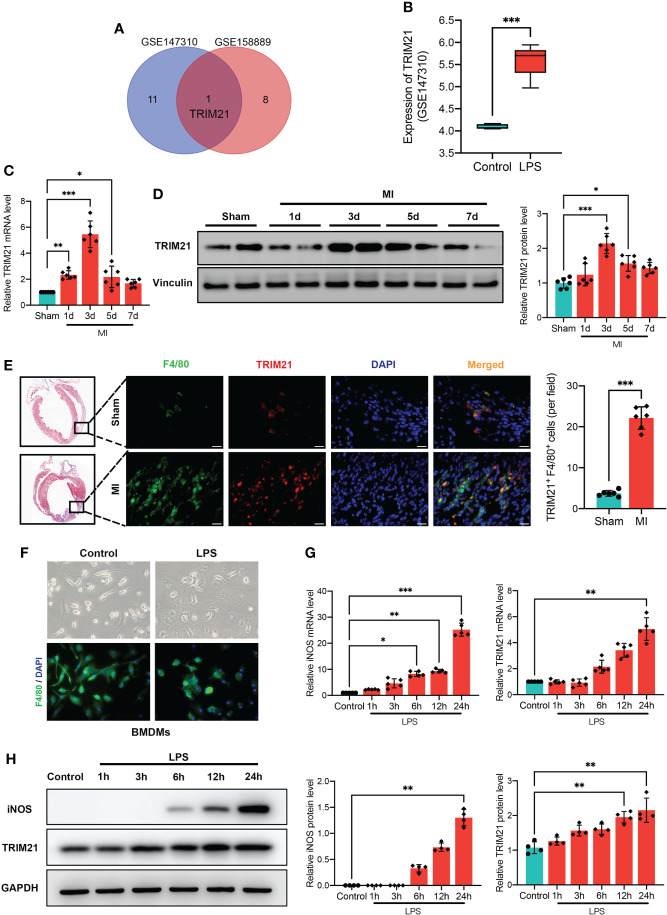
Increased TRIM21 expression after MI and in macrophages by LPS. **(A)** Comparison of upregulated DEGs in GSE147310 and GSE158889 microarray indicates the intersecting gene of TRIM21. **(B)** TRIM21 expression treated with or without LPS in GSE147310. Relative TRIM21 mRNA **(C)** and protein **(D)** levels in the infarcted areas of heart tissues following MI at day 1, 3, 5, and 7, as compared with Sham group. n = 2 mice/group pooled from 3 independent experiments. **(E)** Representative Masson Trichrome staining (left) and immunostaining (right) of TRIM21 (red), F4/80 (green) and DAPI (blue) in the hearts of sham and MI groups (3 days after MI). Scale bar: 20 μm. n = 2 mice/group pooled from 3 independent experiments. **(F)** Representative images of BMDMs stimulated by PBS and LPS. Relative iNOS and TRIM21 mRNA **(G)** and protein **(H)** levels in BMDMs with LPS treatment for 1h, 3h, 6h, 12h, and 24h as compared with control group. Data are representative of 3 independent experiments. n = 3 sets of cells/group. *P<0.05, **P<0.01, and ***P<0.001.

TRIMs family has been proven to mediate the functions of macrophage and inflammatory response (24–28). To this end, we first searched two GEO databases of macrophages stimulated with LPS (**Supplement Figure 1**). We found that the TRIM21 expression was increased in both GEO databases (**Figures 1A, B**). Next, we tested TRIM21 expression in MI tissues. Interestingly, with the permanent ligation of LAD, the mRNA and protein levels of TRIM21 were abundantly expressed in the infarct area on day 3 after MI and decreased to normal levels on day 7 after MI (**Figures 1C, D**). To confirm this finding, we conducted double immunofluorescence staining for TRIM21 and F4/80 (macrophage marker) in the infarcted hearts and observed that TRIM21^+^ F4/80^+^ cells 4- to 5.5-fold higher in the MI group (**Figure 1E**). These observations indicate that TRIM21 might play a role in macrophage-regulated inflammation after MI. To verify this hypothesis, we explored the expression of TRIM21 *in vitro* using BMDMs treated with LPS (**Figure 1F**). After exposure to LPS, inducible nitric oxide synthase (iNOS) levels were gradually increased, suggesting the successful inducement of M1 macrophages. Similarly, the mRNA and protein expression of TRIM21 was also increased in a time-dependent manner (**Figure 1G, H**). These data indicate that the expression of TRIM21 in macrophages is closely associated with MI.

### TRIM21 deficiency improves infarct size and cardiac function after MI

**Figure 2:**
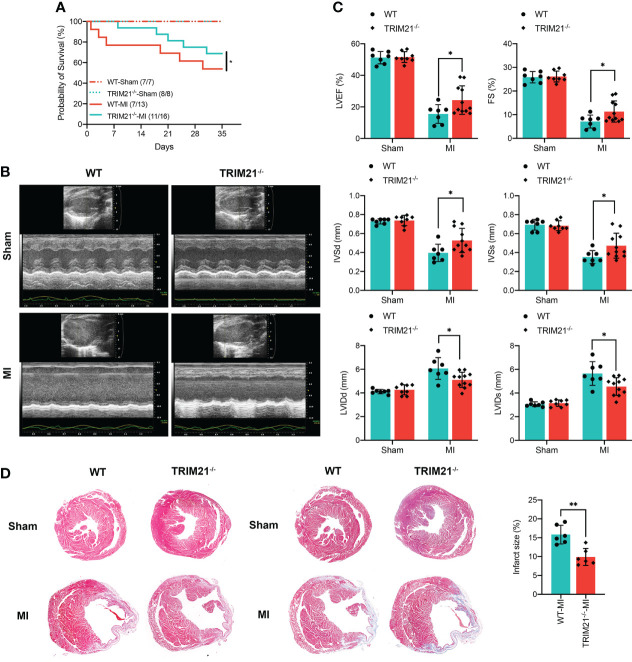
TRIM21 deficiency alleviates MI-induced changes in the cardiac structure and preserves cardiac function. **(A)** Survival rates after MI in WT and TRIM21 knockout (TRIM21^-/-^) mice. **(B)** Representative echocardiographic images of WT mice and TRIM21^-/-^ mice 3 days after sham operation or LAD ligation. **(C)** Quantification of the left ventricular ejection fraction, left ventricular fractional shortening, left ventricular end-systolic internal diameter, and end-diastolic internal diameter. For **(B, C)**, n = 7–11 mice/group pooled from 2 independent experiments. **(D)** Representative H&E staining and Masson Trichrome staining of the cardiac tissue sections of WT mice and TRIM21^-/-^ mice 3 days after sham operation or LAD ligation. Scale bar: 1 mm. Data are representative of 3 independent experiments. n = 2 mice/group. *P<0.05, **P<0.01.

To investigate the function of TRIM21 post-MI, we generated TRIM21 global knockout mice (TRIM21^-/-^) with the TALEN technique (**Supplementary Figure 2A**). The TRIM21 construct interrupted by TALEN was detected with a digestion assay and the cloned polymerase chain reaction (PCR) product sequence. We also used western blot to ensure the successful depletion of TRIM21 in the heart tissues (**Supplement Figures 2B, C**). The mortality in TRIM21^-/-^ mice was significantly reduced than WT mice after LAD ligation, especially from 1 to 7 days. After 5 weeks of MI, 7 of 13 WT mice (54%) versus 11 of 16 TRIM21^-/-^ mice (69%) survived (**Figure 2A**). Measurements of cardiac function were collected for TRIM21^-/-^ and WT mice at 5 weeks after MI. Under normal conditions, loss of TRIM21 did not induce any morphological or functional changes compared to Sham animals (WT-Sham group). However, LVEF in the WT group was severely reduced post-MI, whereas the LVEF in TRIM21^-/-^ mice was significantly preserved. Similarly, the functional deterioration reflected by FS, IVSd, IVSs, LVIDd, and LVIDs was also significantly improved after MI in the TRIM21^-/-^ group compared with WT group (**Figures 2B, C**). Consistent with functional data, TRIM21^-/-^ mice exhibited less irregular morphology, broken fibers, inflammatory cells, and infarct size than WT mice, corroborated by H&E and Masson staining at 35 days in post-MI (**Figure 2D**). These data suggest that TRIM21 is closely associated with cardiac injury in MI and that the absence of TRIM21 improves cardiac function after MI.

### TRIM21 deficiency attenuates cell apoptosis and DNA damage

**Figure 3:**
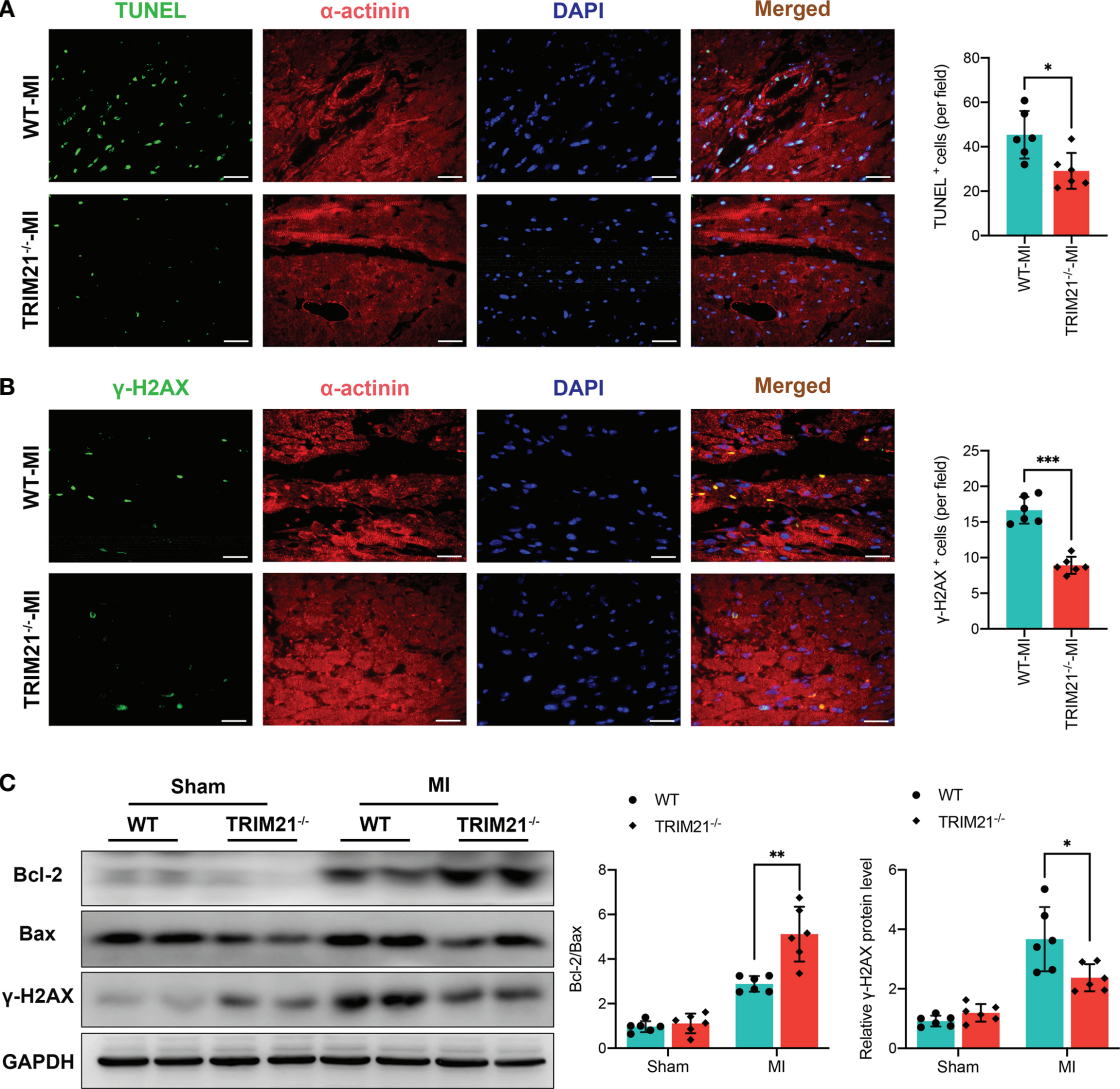
TRIM21 deficiency decreased MI-induced apoptosis and DNA damage. **(A)** Immunofluorescence staining and positive cell-count results of TUNEL in the heart tissues. **(B)** Representative double-fluorescent immunostaining images of γ-H2AX and α-actinin. The positive γ-H2AX (γ-H2AX^+^) cell areas were quantified (right). **(C)** Bcl-2, Bax, and γ-H2AX protein expressions at day 3 post-MI in the infarct zone isolated from WT and TRIM21^-/-^ mice. For A-C, data are representative of 3 independent experiments. n = 2 mice/group. *P<0.05, **P<0.01, and ***P<0.001.

We next explored whether TRIM21 deficiency altered cell apoptosis and DNA damage in the scar areas. TUNEL staining revealed that TRIM21^-/-^ hearts had substantially decreased apoptotic cells on day 3 post-MI (**Figure 3A**). As expected, the ratio of Bcl-2/Bax was markedly exaggerated in TRIM21^-/-^ mice compared to WT hearts in the infarcted area. (**Figure 3C**). Moreover, we observed increased DNA damage and higher protein levels of the DNA damage maker (γ-H2AX) after MI. However, the number of γ-H2AX^+^ cells and the expression of γ-H2AX were lower in the infarcted zone of TRIM21^-/-^ hearts compared to that observed in sham-operated mice (**Figures 3B, C**). These data reveal that TRIM21 deficiency may protect against post-MI-related cell apoptosis and DNA damage processes.

### TRIM21 deficiency suppresses the production of proinflammatory factors and M1 macrophage polarization *in vivo*

**Figure 4:**
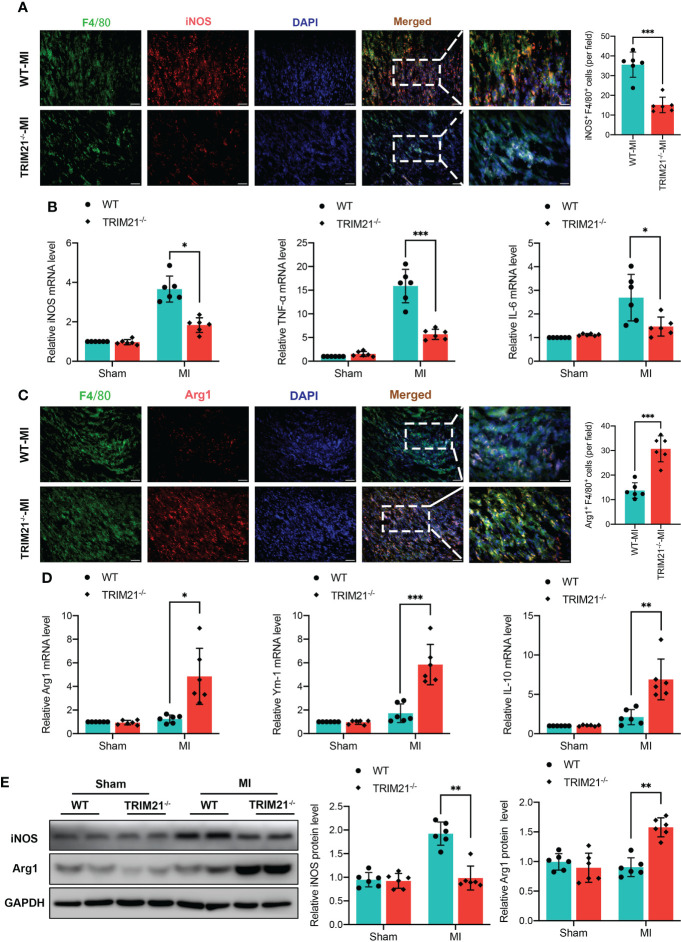
TRIM21 deficiency mediates macrophage polarization in the infarct infarcted zone at day 3 post‐MI. Representative double-fluorescent immunostaining images of F4/80 and iNOS **(A)** and F4/80 and Arg1 **(C)**. The double‐positive iNOS and F4/80 (iNOS^+^/F4/80^+^) areas of M1 macrophages and Arg1 and F4/80 (Arg1^+^/F4/80^+^) M2 macrophages were quantified (right) Scale bar:50 mm. **(B)** Gene expression of iNOS, TNF-α, and IL-6 in heart tissues isolated from infarct border zone at day 3 after MI. n = 2 mice/group pooled from 3 independent experiments. **(D)** Gene expression of Arg1, Ym-1, and IL-10 in heart tissues isolated from infarct zone at day 3 after MI. n = 2 mice/group pooled from 3 independent experiments. **(E)** iNOS and Arg1 protein expression at day 3 post-MI in the infarct zone isolated from WT and TRIM21^-/-^ mice. n = 2 mice/group pooled from 3 independent experiments. *P<0.05, **P<0.01, and ***P<0.001.

To clarify the impact of TRIM21 on macrophage polarization, we analyzed the expression of macrophage phenotype markers in TRIM21^-/-^ hearts. The number of F4/80^+^iNOS^+^ cells was significantly attenuated in the TRIM21-/- mice as compared with WT-MI hearts (**Figure 4A**). In contrast, F4/80^+^Arg1^+^ M2 macrophages were increased in the TRIM21^-/-^ group compared with WT mice post-MI (**Figure 4C**). Moreover, the mRNA levels of M1 biomarkers iNOS, TNF-α, and IL-6 were significantly decreased in TRIM21^-/-^ mice, while the levels of M2 biomarkers Arg1, Ym-1, and IL-10 were markedly increased in TRIM21^-/-^ compared with WT mice on day 3 post-MI (**Figures 4B, D**). Similar protein expression patterns of iNOS and Arg1 were observed in the WT and TRIM21^-/-^ heart tissues post-MI (**Figure 4E**). These data indicate that TRIM21 deficiency accelerates M2 macrophage polarization with reduced M1 polarization in post-MI hearts.

### Effects of TRIM21 overexpression on macrophage polarization *in vitro*

**Figure 5:**
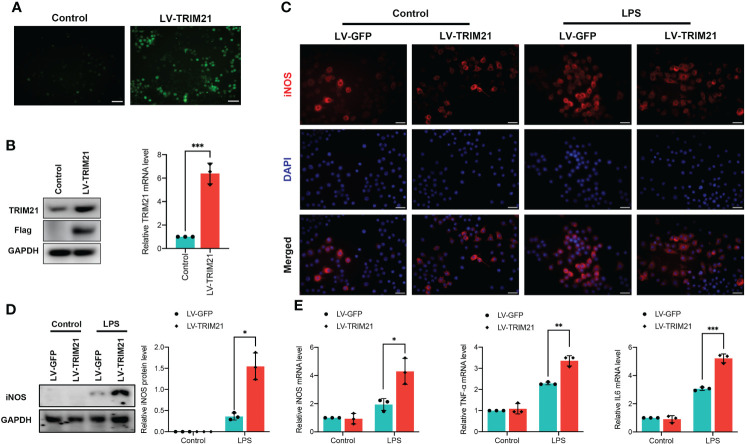
TRIM21 overexpression induces macrophage polarized into M1 phenotype in RAW264.7 cells. **(A)** Representative images of RAW264.7 cells transfected with lentivirus targeting the TRIM21 gene (LV-TRIM21) or negative lentivirus (LV-GFP). The protein levels of TRIM21 and Flag **(A)** and the mRNA of TRIM21 **(B)** in RAW264.7 cells. **(C)** Representative images of iNOS staining in LPS-stimulated RAW264.7 cells transfected with LV-TRIM21 or LV-GFP. Scale bar: 200 mm. **(D)** The protein level of iNOS and mRNA levels of iNOS, IL-6 and TNF-α **(E)** in LPS-stimulated RAW264.7 cells transfected with LV-TRIM21 or LV-GFP. For **(A-E)**, data are representative of 3 independent experiments. n = 3 sets of cells/group. *P<0.05, **P<0.01, and ***P<0.001.

To determine the molecular link between TRIM21 and macrophage polarization in more detail, we subjected TRIM21 lentivirus (LV-TRIM21) to overexpress TRIM21 in LPS-stimulated RAW264.7 macrophages (**Figure 5A**). Western blot and qRT-PCR confirmed that LV-TRIM21 transfection significantly increased the expression of TRIM21 (**Figure 5B**). As shown in **Figure 5C**, LPS elicited upregulation of iNOS^+^ cells in LV-GFP-stimulated RAW264.7 was significantly increased by TRIM21 overexpression. Similarly, LPS stimulation markedly increased the protein levels of iNOS, and TRIM21 overexpression significantly promoted iNOS levels compared with the LV-GFP-transfected controls (**Figure 5D**). Contrary to the observation in the TRIM21^-/-^ mice, TRIM21 overexpression significantly increased the mRNA levels of M1 biomarkers iNOS, TNF-α, and IL-6 (**Figure 5E**) with LPS exposure. Therefore, these findings suggest that TRIM21 is responsible for the M1 polarization of macrophages.

### TRIM21 regulates the PI3K/Akt pathway *in vivo* and *in vitro*

**Figure 6:**
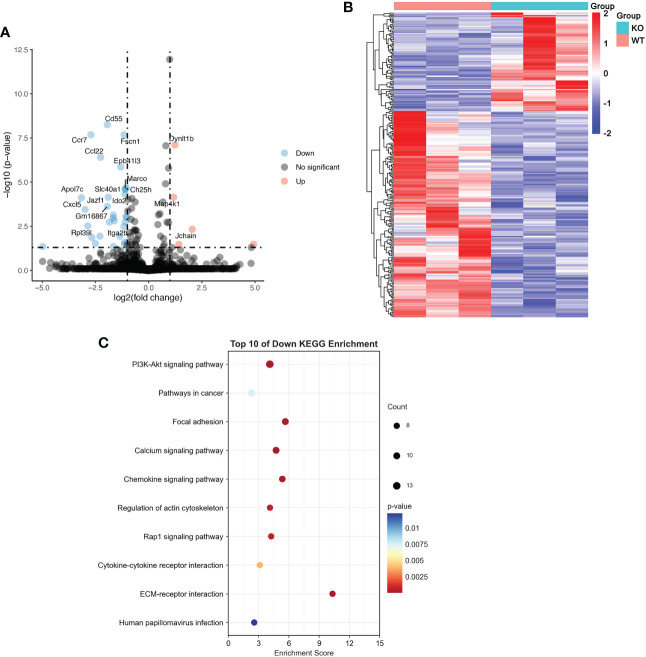
RNA-seq results of LPS-treated BMDMs with or without TRIM21 depletion **(A)** Volcano plot of DEGs between WT and TRIM21^-/-^ in BMDMs treated with LPS for 12 h. Red color indicates genes that are upregulated and blue color indicates downregulated genes. **(B)** Heatmap of color-coded expression levels of DEGs. **(C)** Top 10 KEGG down regulated signaling pathways.

To verify the exact mechanism of TRIM21 in macrophage polarization, we performed RNA-seq of LPS-stimulated BMDMs isolated from WT and TRIM21^-/-^ mice. As shown in **Figure 6A**, TRIM21 deficiency increased 81 genes and decreased 170 genes in LPS-treated BMDMs compared to the WT group. The DEGs between the control and LPS group is presented as a heatmap in **Figure 6B**. KEGG function analysis indicated that differentially down-regulated genes were highly enriched in the PI3K-Akt signaling pathway, pathways in cancer, focal adhesion, calcium signaling pathway, regulation of actin cytoskeleton, Rap1 signaling pathway, cytokine and cytokine receptor, ECM-receptor interaction, and human papillomavirus infection (**Figure 6C**). Concerning that the PI3K-Akt signaling pathway is associated with M1 macrophage polarization and TRIM21 expression (29, 30), we then focused on the PI3K-Akt signaling pathway.

**Figure 7:**
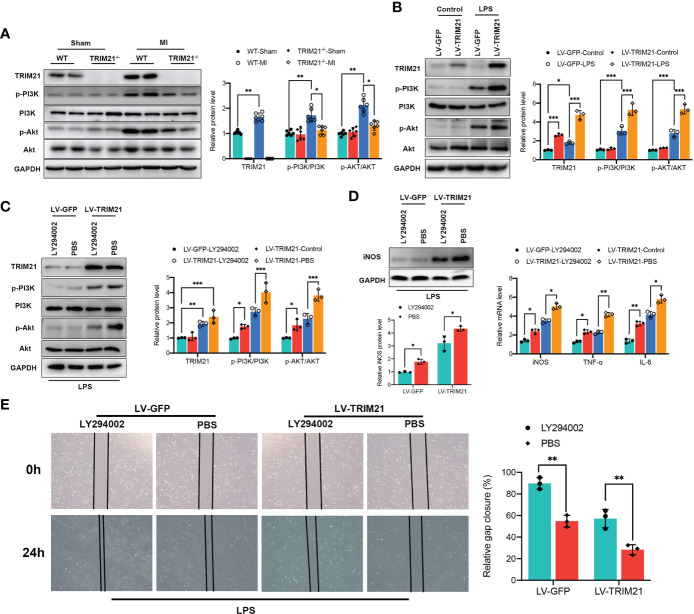
TRIM21 regulates macrophage polarization by the PI3K/Akt pathway. **(A)** The protein levels of TRIM21, p-PI3K, PI3K, p-AKT and AKT on day 3 post-MI in the infarct zone isolated from WT and TRIM21^-/-^ mice. **(B)** The protein levels of TRIM21, p-PI3K, PI3K, p-AKT and AKT in LPS-stimulated RAW264.7 cells transfected with LV-GFP or LV-TRIM21, **(C)** and in LV-GFP and LV-TRIM21 group treated with LY294002 or PBS after LPS exposure. **(D)** The protein levels of iNOS (left) and mRNA levels of iNOS, TNF-α, and IL-6 (right) in LV-GFP and LV-TRIM21 group treated with LY294002 or PBS. **(E)** Representative images and quantified results of gap closure of cardiac fibroblast cultured in conditioned medium from LV-GFP and LV-TRIM21 macrophages treated with LY294002 or PBS macrophages after LPS exposure. For **(A-E)**, data are representative of 3 independent experiments. n = 3 sets of cells/group. *P<0.05, **P<0.01, and ***P<0.001.

We first investigated the expression of the PI3K/Akt pathway *in vivo*. Expression of p-PI3K and p-Akt protein was significantly increased at 3 days after MI, whereas TRIM21 depletion suppressed p-PI3K and p-Akt levels in MI hearts (**Figure 7A**). Next, we examined the levels of p-PI3K and p-AKT in RAW264.7 cells treated with LPS. In contrast to observation *in vivo*, TRIM21 overexpression further increased the levels of p-PI3K and p-Akt with LPS stimulation compared with LV-GFP-transfected controls (**Figure 7B**). We further used a PI3K inhibitor, LY294002, to suppress the expression of PI3K and p-PI3K. Treatment with LY294002 reversed the elevated p-PI3K and p-Akt levels induced by TRIM21 overexpression (**Figure 7C**). In line with this, M1 macrophage polarization marker levels induced by TRIM21 overexpression were eliminated with the inhibition of P13K (**Figure 7D**). On a functional level, we performed a wound-healing scratch assay to assess the effects of TRIM21 overexpression in RAW264.7 cells on the migration of cardiac fibroblasts. Macrophages were exposed to LPS after TRIM21 overexpression with or without PI3K inhibitor for 12 h, and the conditioned medium was collected. Notably, conditioned medium from TRIM21-overexpression RAW264.7 cells decreased the effects in stimulating cardiac fibroblast migration. Blocking PI3K by adding PI3K inhibitor to the culture medium alleviated the function of TRIM21 overexpression (**Figure 7E**). Therefore, these data indicate that TRIM21 promotes M1 macrophage polarization and macrophage-related function partly *via* the PI3K/Akt pathway.

## Discussion

During the MI process, a host of M1 macrophages infiltrate into infarcted areas and provoke the initial inflammatory response. Appropriate inflammation after MI is critical for heart repair. However, an excessive inflammatory response collectively inflicts damage on cardiac remodeling. We present evidence that implicates the effects of TRIM21 in macrophage polarization post-MI. We determine that TRIM21expression peaks in the infarcted heart on day 3 after MI. Loss of TRIM21 protected the heart from MI-related cardiac dysfunction, cell apoptosis, DNA damage, and inflammatory response. Instead, inhibition of PI3K/Akt pathway alleviates M1 phenotype in TRIM21 overexpression macrophages, suggesting that TRIM21 influence macrophage polarization by the PI3K/Akt pathway after MI.

Tripartite motif-containing proteins (TRIMs) are a large family of E3 ubiquitin ligases containing three highly conserved domains, namely a RING (Really Interesting New Gene) finger, one or two B -box domains, and a coiled-coil domain (31). TRIMs family contains more than 70 members and participates in multiple cellular cascades, including the immunity response, tumor progression, and autophagy (14, 32–34). Recently, the effects of TRIMs on myocardial ischemia were explored. For example, the upregulation of TRIM44 by non‐coding RNA hypoxia‐inducible factor 1α (HIF1A) ‐antisense RNA 2 (HIF1A‐AS2) is associated with cardiac cell apoptosis, migration, and invasion in hypoxia-treated human cardiomyocytes (35). TRIM72 levels are increased in MI and are positively related to Global Registry of Acute Coronary Events (GRACE) scores (36). Moreover, TRIM72 deficiency exacerbates ischemia-reperfusion injury (I/R)-induced myocardial damage, and overexpression of TRIM72 alleviates cardiac dysfunction after MI (37, 38). TRIM33 aggravates I/R-related reactive oxygen species (ROS) and cardiac damage by promoting glutathione peroxidase 1 (glutathione peroxidase 1, GPX1) ubiquitination and proteasome-dependent degradation (39). However, the role of TRIMs family in regulating macrophage polarization after MI remains unclear.

Cardiomyocyte apoptosis is a major contributor to cardiac dysfunction after MI (40–42). TRIM21 levels are increased in various diseases and are associated with cell apoptosis. For instance, enhanced TRIM21 expression was found in HT22 hippocampal neurons with oxygen-glucose deprivation/reoxygenation (OGD/R) stimulation (43). Depletion of TRMI21 alleviated OGD/R-induced cell apoptosis, inflammation, and oxidative stress in HT22 cells. We speculated whether TRIM21 could mediate the cell apoptosis process in the heart. As confirmed by TUNEL staining, cell apoptosis in TRIM21^-/-^ mice were dramatically reduced compared with that in WT mice. This was also determined by the increased Bcl-2/Bax protein ratio. In addition, MI induces reactive oxygen species (ROS) production and DNA damage, which culminates in cell necrosis and inflammatory response (44, 45). In the present study, we found that DNA damage in the infarcted heart of TRIM21^-/-^ mice was significantly decreased after MI, as indicated by the DNA damage maker, γ-H2AX. Consistent with our findings, it was also proven that γ-H2AX was reduced after TAC in TRIM21^-/-^ mice, which indicated attenuation of oxidative stress by TRIM21 loss (16). Guha et al. identified that UV radiation-induced DNA damage enhanced TRIM21 expression, which further triggered the proteasomal degradation of HuR (46, 47). With the development of cell apoptosis and DNA damage after MI, cardiac structure and function were disrupted. In line with these findings, we revealed that TRIM21 depletion improves scar formation, cardiac function, and mouse survival. These data indicate that TRIM21 is an essential regulator after MI.

Macrophage polarization is a critical but poorly understood process in the proinflammatory phase of MI (48). Following MI, the depletion of macrophages causes increased necrotic cells, neutrophil accumulation, and reduced collagen deposition, leading to infarct expansion (49, 50). For instance, interruption of C-C chemokine receptor 2 (CCR2), AXL receptor tyrosine kinase, and small mothers against decapentaplegic 3(Smad3) signaling delayed macrophage polarization from M1 to M2 phenotype macrophages, resulting in an excessive inflammatory response and adverse ventricular remodeling (9, 10, 51). TRIM21(Ro52/SS-A) is a target autoantigen that plays a critical role in several systemic autoimmune diseases, infectious diseases, and viral-induced cardiac injuries (8, 14, 34, 52). Recently, Brauner et al. found that TRIM21 was associated with the stability of atherosclerotic plaques (53). TRMI21^-/-^ mice fed a high-fat diet developed larger plaques with more clotting substances, which further increased the stability of the plaque. Moreover, TRIM21 is more abundantly expressed in macrophages than that in monocytes, suggesting that it may play a critical role in initiating the proinflammatory response in macrophages (54). Similarly, we determined that the expression of TRIM21 in macrophages was markedly increased and peaked at 3 days after MI. Bioinformatics analysis from human MDMs and mice BMDMs is consistent with our findings. Consistent with these results, toll-like receptor 3 (TLR3) and TLR4 ligands can induce the upregulation of TRIM21 in human THP1-derived macrophages (55). Indeed, TRIM21 deficiency results in decreased proinflammatory macrophages and subsequent accumulation of reparative phenotype macrophages in infarcted hearts. As expected, the overexpression of TRIM21 established a conversed result *in vitro*. Consistent with our results, the expression of iNOS and IL6 in BMDMs derived from TRIM21^-/-^ mice was also significantly reduced (17). Similarly, inhibition of TRIM21 inhibited M1polarization in LPS-stimulated BV2 microglia cells (56). These data indicate that TRIM21 exerts a substantial impact on macrophage polarization after MI.

**Figure 8:**
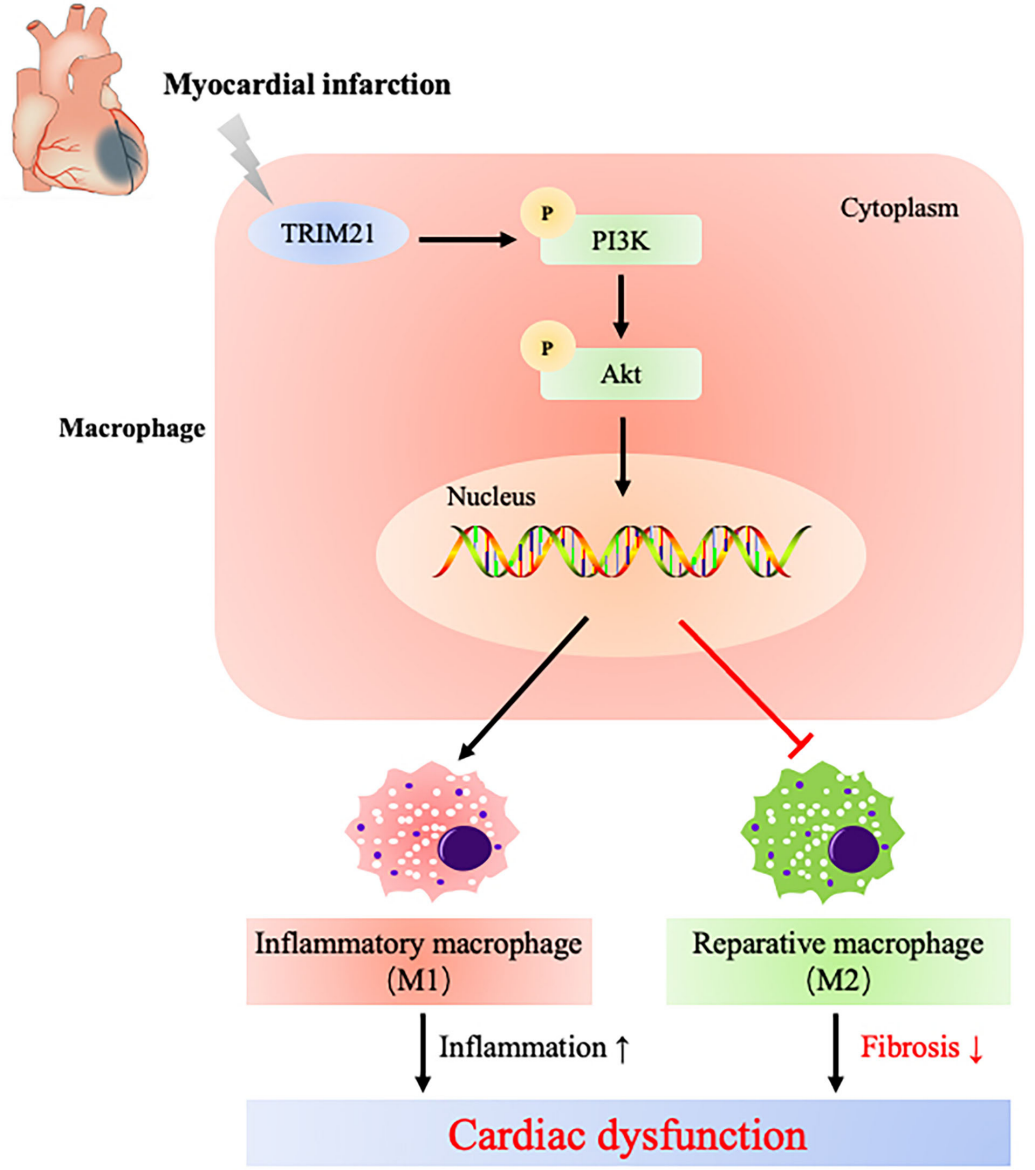
Schematic diagram illustrates that TRIM21 mediates macrophage polarization *via* PI3K/Akt pathway after myocardial infarction.

We further uncover that TRIM21-regulated macrophage polarization might be due to the PI3K/Akt pathway by RNA-Seq (**Figure 8**). The PI3K/Akt pathway plays a vital role in regulating macrophage survival, migration, and proliferation (57–59). Phosphatase and tensin homolog (PTEN) suppress PI3K activation by converting phosphatidylinositol-3,4-triphosphate (PIP_3_) into phosphatidylinositol-3,4-biphosphate (PIP_2_), and PTEN absence increases PI3K/Akt activity and inhibits LPS-induced macrophage response (60). Moreover, the relationship between TRIM21 and PI3K/Akt pathway has been proven before. Lee et al. confirmed that Akt phosphorylated PFKP and suppressed TRIM21-mediated degradation of PFKP, which promoted cancer progression (61). Our results show that the ratio of p-PI3k/PI3K and p-Akt/Akt increased in LPS-stimulated macrophages, which was further increased by overexpressing TRIM21. Similarly, the depletion of TRIM21 decreased the expression of p-PI3k and Akt. Furthermore, we found that LY294002, a PI3K-specific inhibitor, downregulated iNOS expression induced by TRIM21 overexpression, consistent with the results reported in previous studies (62, 63). These data indicate that TRIM21 regulates M1 polarization of macrophages at least partly *via* the P13K/Akt pathway.

In conclusion, our results suggest that TRIM21 is an upstream regulator of macrophage polarization into the M1 phenotype *via* the PI3K/Akt signaling pathway after MI, which provides a novel therapeutic target in treating post-MI-related inflammatory injury.

## Limitations

In the present study, we determined how TRIM21 regulates proinflammatory macrophage function post-MI. First, concerning that the process of inflammation is complex and varied and we performed TRIM21 loss in global KO mice, the interaction between TRIM21 in other inflammatory cells and post-MI response in the infarct area was not discussed. For example, TRIM21 depletion can promote Th17 differentiation, resulting in stable atherosclerotic plaques (53). Then, macrophage polarization might be regulated by numerous signaling pathways. Based on the results of RNA-seq, we determined several signaling pathways that might involve in the process of TRIM21-regulated macrophage polarization. However, our study only focused on the role of the PI3K/Akt pathway, the mechanisms of TRIM21 in macrophage function and polarization by other pathways in the heart remain further explored.

## Data availability statement

Publicly available datasets were analyzed in this study. This data can be found here: RNA-seq data were deposited in GEO (GSE214696). All data that support the findings of the present study are available from the corresponding author upon reasonable request.

## Ethics statement

The animal study was reviewed and approved by Ethics Committee of Shanghai Tenth People’s Hospital of Tongji University School of Medicine.

## Author contributions

ZL: Data curation, investigation, writing—original draft. XL: Investigation, methodology, validation. XZ: Methodology. WZ: Methodology, validation. MG: Data curation. XQ: Methodology. JL: Data curation, project administration. YF: Software. BL: Funding acquisition. YW: Funding acquisition, writing—review and editing, supervision. All authors contributed to the article and approved the submitted version.

## Funding

This work was supported by the Natural Science Foundation of Shanghai [Grant No.18ZR1429700] and the National Natural Science Foundation of China [Grant No.81270193] to YW, the Shanghai Sailing Program [No. 19YF1437900], the Climbing Program of Shanghai Tenth People’s Hospital [No. 2021SYPDRC035] and the National Natural Science Foundation of China [No. 81900385] to BL.

## Acknowledgments

We would like to thank Zhanju Liu (Department of Gastroenterology, Shanghai Tenth People’s Hospital of Tongji University School of Medicine) for providing TRIM21 knockout mice for us.

## Conflict of interest

The authors declare that the research was conducted in the absence of any commercial or financial relationships that could be construed as a potential conflict of interest.

## Publisher’s note

All claims expressed in this article are solely those of the authors and do not necessarily represent those of their affiliated organizations, or those of the publisher, the editors and the reviewers. Any product that may be evaluated in this article, or claim that may be made by its manufacturer, is not guaranteed or endorsed by the publisher.

## Supplementary material

Supplementary Figure 1 Differentially expressed of TRIMs family in LPS-stimulated macrophages. Heatmap shows the log10 (normalized counts +0.01) of TRIMs family from **(A)** GSE147310 and **(B)** GSE158889 microarray. Top 25 differentially expressed genes (DEG) in **(C)** GSE147310 and **(D)** GSE158889 microarray. Red represents high expression, blue means low expression.

Supplementary Figure 2 Establishment and verification of TRIM21 knockout in mice. **(A)** Diagram of the partial TRIM21 gene body showing the TALEN (Transcription Activator-Like Effector Nuclease) target sites (top), and DNA sequences of the cloned polymerase chain reaction products in WT, TRIM21^-/-^ and TRIM21 heterozygous (TRM21^+/-^) mice (bottom). **(B)** Agarose gel images of digestion products from WT and TRIM21KO mouse tail digested with a T7E1 assay. **(C)** Western blot assay shows the level of the TRIM21 protein in the WT and TRIM21^-/-^ hearts. Data are representative of 3 independent experiments. n = 2 mice/group.

## References

[1] Sandoval Y Jaffe AS. Type 2 myocardial infarction: JACC review topic of the week. J Am Coll Cardiol (2019) 73:1846–60. doi: 10.1016/j.jacc.2019.02.018 30975302

[2] Sabatine MS. Differentiating type 1 and type 2 myocardial infarction: Unfortunately, still more art than science. JAMA Cardiol (2021) 6:781. doi: 10.1001/jamacardio.2021.0693 33881457

[3] Hofmann R James SK Jernberg T Lindahl B Erlinge D Witt N. Oxygen therapy in suspected acute myocardial infarction. N Engl J Med (2017) 377:1240–9. doi: 10.1056/NEJMoa1706222 28844200

[4] Uriel N Sayer G Annamalai S Kapur NK Burkhoff D. Mechanical unloading in heart failure. J Am Coll Cardiol (2018) 72:569–80. doi: 10.1016/j.jacc.2018.05.038 30056830

[5] Lambert JM Lopez EF Lindsey ML. Macrophage roles following myocardial infarction. Int J Cardiol (2008) 130:147–58. doi: 10.1016/j.ijcard.2008.04.059 PMC2857604 18656272

[6] Murray PJ Allen JE Biswas SK Fisher EA Gilroy DW Goerdt S. Macrophage activation and polarization: nomenclature and experimental guidelines. Immunity (2014) 41:14–20. doi: 10.1016/j.immuni.2014.06.008 25035950 PMC4123412

[7] Fujiu K Wang J Nagai R. Cardioprotective function of cardiac macrophages. Cardiovasc Res (2014) 102:232–9. doi: 10.1093/cvr/cvu059 24675722

[8] Jia D Jiang H Weng X Wu J Bai P Yang W. Interleukin-35 promotes macrophage survival and improves wound healing after myocardial infarction in mice. Circ Res (2019) 124:1323–36. doi: 10.1161/CIRCRESAHA.118.314569 30832557

[9] Palevski D Levin-Kotler LP Kain D Naftali-Shani N Landa N Ben-Mordechai T. Loss of macrophage wnt secretion improves remodeling and function after myocardial infarction in mice. J Am Heart Assoc (2017) 6:e004387–e004405. doi: 10.1161/JAHA.116.004387 PMC5523630 28062479

[10] DeBerge M Glinton K Subramanian M Wilsbacher LD Rothlin CV Tabas I. Macrophage AXL receptor tyrosine kinase inflames the heart after reperfused myocardial infarction. J Clin Invest (2021) 131:e139576–e139590. doi: 10.1172/JCI139576 PMC7954597 33529176

[11] Lorchner H Poling J Gajawada P Hou Y Polyakova V Kostin S. Myocardial healing requires Reg3beta-dependent accumulation of macrophages in the ischemic heart. Nat Med (2015) 21:353–62. doi: 10.1038/nm.3816 25751817

[12] Alomari M. TRIM21 - a potential novel therapeutic target in cancer. Pharmacol Res (2021) 165:105443. doi: 10.1016/j.phrs.2021.105443 33508433

[13] Zhang Z Bao M Lu N Weng L Yuan B Liu YJ. The E3 ubiquitin ligase TRIM21 negatively regulates the innate immune response to intracellular double-stranded DNA. Nat Immunol (2013) 14:172–8. doi: 10.1038/ni.2492 PMC3645272 23222971

[14] Hos NJ Fischer J Hos D Hejazi Z Calabrese C Ganesan R. TRIM21 is targeted for chaperone-mediated autophagy during salmonella typhimurium infection. J Immunol (2020) 205:2456–67. doi: 10.4049/jimmunol.2000048 PMC7576115 32948684

[15] Zhou G Wu H Lin J Lin R Feng B Liu Z. TRIM21 is decreased in colitis-associated cancer and negatively regulates epithelial carcinogenesis. Inflammation Bowel Dis (2020) 458–468. doi: 10.1093/ibd/izaa229 32860065

[16] Pan JA Sun Y Jiang YP Bott AJ Jaber N Dou Z. TRIM21 ubiquitylates SQSTM1/p62 and suppresses protein sequestration to regulate redox homeostasis. Mol Cell (2016) 61:720–33. doi: 10.1016/j.molcel.2016.02.007 PMC4779181 26942676

[17] Sjostrand M Carow B Nyberg WA Covacu R Rottenberg ME Espinosa A. TRIM21 controls toll-like receptor 2 responses in bone-marrow-derived macrophages. Immunology (2020) 159:335–43. doi: 10.1111/imm.13157 PMC7011629 31755557

[18] Song R Gao Y Dozmorov I Malladi V Saha I McDaniel MM. IRF1 governs the differential interferon-stimulated gene responses in human monocytes and macrophages by regulating chromatin accessibility. Cell Rep (2021) 34:108891. doi: 10.1016/j.celrep.2021.108891 33761354 PMC8300000

[19] Mia MM Cibi DM Abdul Ghani SAB Song W Tee N Ghosh S. YAP/TAZ deficiency reprograms macrophage phenotype and improves infarct healing and cardiac function after myocardial infarction. PloS Biol (2020) 18:e3000941. doi: 10.1371/journal.pbio.3000941 33264286 PMC7735680

[20] Zhou G Wu W Yu L Yu T Yang W Wang P. Tripartite motif-containing (TRIM) 21 negatively regulates intestinal mucosal inflammation through inhibiting TH1/TH17 cell differentiation in patients with inflammatory bowel diseases. J Allergy Clin Immunol (2018) 142:1218–1228.e1212. doi: 10.1016/j.jaci.2017.09.038 29113905

[21] Rienks M Carai P Bitsch N Schellings M Vanhaverbeke M Verjans J. Sema3A promotes the resolution of cardiac inflammation after myocardial infarction. Basic Res Cardiol (2017) 112:42. doi: 10.1007/s00395-017-0630-5 28540528 PMC5443852

[22] Toda G Yamauchi T Kadowaki T Ueki K. Preparation and culture of bone marrow-derived macrophages from mice for functional analysis. STAR Protoc (2021) 2:100246. doi: 10.1016/j.xpro.2020.100246 33458708 PMC7797923

[23] Pfaffl MW. A new mathematical model for relative quantification in real-time RT-PCR. Nucleic Acids Res (2001) 29:e45. doi: 10.1093/nar/29.9.e45 11328886 PMC55695

[24] Jin Z Zhu Z Zhang W Liu L Tang M Li D. Effects of TRIM59 on RAW264.7 macrophage gene expression and function. Immunobiology (2021) 226:152109. doi: 10.1016/j.imbio.2021.152109 34252840

[25] Lou J Wang Y Zheng X Qiu W. TRIM22 regulates macrophage autophagy and enhances mycobacterium tuberculosis clearance by targeting the nuclear factor-multiplicity κB/beclin 1 pathway. J Cell Biochem (2018) 119:8971–80. doi: 10.1002/jcb.27153 30011088

[26] Tokarz Debra A Heffelfinger Amy K Jima Dereje D Jamie G Shah Radhika N Rodriguez-Nunez I. Disruption of Trim9 function abrogates macrophage motility *in vivo* . J Leukocyte Biol (2017) 102:1371–80. doi: 10.1189/jlb.1A0816-371R PMC6608060 29021367

[27] Lu M Zhu X Yang Z Zhang W Sun Z Ji Q. E3 ubiquitin ligase tripartite motif 7 positively regulates the TLR4-mediated immune response *via* its E3 ligase domain in macrophages. Mol Immunol (2019) 109:126–33. doi: 10.1016/j.molimm.2019.01.015 30928727

[28] Gallouet A-S Ferri F Petit V Parcelier A Lewandowski D Gault N. Macrophage production and activation are dependent on TRIM33. Oncotarget (2017) 8:5111–22. doi: 10.18632/oncotarget.13872 PMC5354896 27974684

[29] Huang Y He S Chen Y Sheng J Fu Y Du X. UCHL1 promoted polarization of M1 macrophages by regulating the PI3K/AKT signaling pathway. J Inflammation Res (2022) 15:735–46. doi: 10.2147/JIR.S343487 PMC8824699 35153498

[30] Cheng J Huang Y Zhang X Yu Y Wu S Jiao J. TRIM21 and PHLDA3 negatively regulate the crosstalk between the PI3K/AKT pathway and PPP metabolism. Nat Commun (2020) 11:1880. doi: 10.1038/s41467-020-15819-3 32312982 PMC7170963

[31] Borlepawar A Frey N Rangrez AY. A systematic view on E3 ligase ring TRIMmers with a focus on cardiac function and disease. Trends Cardiovasc Med (2019) 29:1–8. doi: 10.1016/j.tcm.2018.05.007 29880235

[32] Dai W Wang J Wang Z Xiao Y Li J Hong L. Comprehensive analysis of the prognostic values of the TRIM family in hepatocellular carcinoma. Front Oncol (2021) 11:767644. doi: 10.3389/fonc.2021.767644 35004288 PMC8733586

[33] Pu C Ciren Y Liu Y Long Z. TRIM52 knockdown inhibits cell proliferation and induces apoptosis through activation of the STAT3 pathway in ovarian cancer1. All Life (2021) 14:646–56. doi: 10.1080/26895293.2021.1947394

[34] Liu H Li M Song Y Xu W. TRIM21 restricts coxsackievirus B3 replication, cardiac and pancreatic injury *via* interacting with MAVS and positively regulating IRF3-mediated type-I interferon production. Front Immunol (2018) 9:2479. doi: 10.3389/fimmu.2018.02479 30410495 PMC6209670

[35] Luo F Wu Y Zhu L Zhang J Liu Y Jia W. Knockdown of HIF1A-AS2 suppresses TRIM44 to protect cardiomyocytes against hypoxia-induced injury. Cell Biol Int (2020) 44:1523–34. doi: 10.1002/cbin.11348 32222118

[36] Xie H Wang Y Zhu T Feng S Yan Z Zhu Z. Serum MG53/TRIM72 is associated with the presence and severity of coronary artery disease and acute myocardial infarction. Front Physiol (2020) 11:617845. doi: 10.3389/fphys.2020.617845 33391037 PMC7773634

[37] Shan D Guo S Wu HK Lv F Jin L Zhang M. Cardiac ischemic preconditioning promotes MG53 secretion through H2O2-activated protein kinase c-delta signaling. Circulation (2020) 142:1077–91. doi: 10.1161/CIRCULATIONAHA.119.044998 32677469

[38] Feng H Shen H Robeson MJ Wu YH Wu HK Chen GJ. MG53 E3 ligase-dead mutant protects diabetic hearts from acute Ischemic/Reperfusion injury and ameliorates diet-induced cardiometabolic damage. Diabetes (2022) 71:298–314. doi: 10.2337/db21-0322 34844991 PMC8914286

[39] Jian Z Liang B Pan X Xu G Guo SS Li T. CUEDC2 modulates cardiomyocyte oxidative capacity by regulating GPX1 stability. EMBO Mol Med (2016) 8:813–29. doi: 10.15252/emmm.201506010 PMC4931293 27286733

[40] Jose Corbalan J Vatner DE Vatner SF. Myocardial apoptosis in heart disease: does the emperor have clothes? Basic Res Cardiol (2016) 111:31. doi: 10.1007/s00395-016-0549-2 27043720

[41] Wang X Guo Z Ding Z Mehta JL. Inflammation, autophagy, and apoptosis after myocardial infarction. J Am Heart Assoc (2018) 7:e008024–e008037. doi: 10.1161/JAHA.117.008024 PMC6015297 29680826

[42] Heusch G. Myocardial ischaemia-reperfusion injury and cardioprotection in perspective. Nat Rev Cardiol (2020) 17:773–89. doi: 10.1038/s41569-020-0403-y 32620851

[43] Fu Y Gao J Li Y Yang X Zhang Y. TRIM21 deficiency confers protection from OGD/R-induced oxidative and inflammatory damage in cultured hippocampal neurons through regulation of the Keap1/Nrf2 pathway. Int Immunopharmacol (2022) 103:108414. doi: 10.1016/j.intimp.2021.108414 34929478

[44] Shahzad S Hasan A Faizy AF Mateen S Fatima N Moin S. Elevated DNA damage, oxidative stress, and impaired response defense system inflicted in patients with myocardial infarction. Clin Appl Thromb Hemost (2018) 24:780–9. doi: 10.1177/1076029617725602 PMC6714874 28946755

[45] Bhat MA Gandhi G. Elevated oxidative DNA damage in patients with coronary artery disease and its association with oxidative stress biomarkers. Acta Cardiol (2019) 74:153–60. doi: 10.1080/00015385.2018.1475093 29914299

[46] Guha A Ahuja D Das Mandal S Parasar B Deyasi K Roy D. Integrated regulation of HuR by translation repression and protein degradation determines pulsatile expression of p53 under DNA damage. iScience (2019) 15:342–59. doi: 10.1016/j.isci.2019.05.002 PMC6548907 31103853

[47] Guha A Nag S Ray PS. Negative feedback regulation by HuR controls TRIM21 expression and function in response to UV radiation. Sci Rep (2020) 10:11753–11767. doi: 10.1038/s41598-020-68646-3 PMC7367240 32678213

[48] Mouton AJ DeLeon-Pennell KY Rivera Gonzalez OJ Flynn ER Freeman TC Saucerman JJ. Mapping macrophage polarization over the myocardial infarction time continuum. Basic Res Cardiol (2018) 113:26. doi: 10.1007/s00395-018-0686-x 29868933 PMC5986831

[49] Haider N Bosca L Zandbergen HR Kovacic JC Narula N Gonzalez-Ramos S. Transition of macrophages to fibroblast-like cells in healing myocardial infarction. J Am Coll Cardiol (2019) 74:3124–35. doi: 10.1016/j.jacc.2019.10.036 PMC7425814 31856969

[50] de Couto G Liu W Tseliou E Sun B Makkar N Kanazawa H. Macrophages mediate cardioprotective cellular postconditioning in acute myocardial infarction. J Clin Invest (2015) 125:3147–62. doi: 10.1172/JCI81321 PMC4563759 26214527

[51] Chen B Huang S Su Y Wu YJ Hanna A Brickshawana A. Macrophage Smad3 protects the infarcted heart, stimulating phagocytosis and regulating inflammation. Circ Res (2019) 125:55–70. doi: 10.1161/CIRCRESAHA.119.315069 31092129 PMC6681442

[52] Espinosa A Dardalhon V Brauner S Ambrosi A Higgs R Quintana FJ. Loss of the lupus autoantigen Ro52/Trim21 induces tissue inflammation and systemic autoimmunity by disregulating the IL-23-Th17 pathway. J Exp Med (2009) 206:1661–71. doi: 10.1084/jem.20090585 PMC2722164 19635858

[53] Brauner S Jiang X Thorlacius GE Lundberg AM Ostberg T Yan ZQ. Augmented Th17 differentiation in Trim21 deficiency promotes a stable phenotype of atherosclerotic plaques with high collagen content. Cardiovasc Res (2018) 114:158–67. doi: 10.1093/cvr/cvx181 29016728

[54] Lenart M Rutkowska-Zapala M Szatanek R Weglarczyk K Stec M Bukowska-Strakova K. Alterations of TRIM21-mRNA expression during monocyte maturation. Immunobiology (2017) 222:494–8. doi: 10.1016/j.imbio.2016.10.016 27773663

[55] Jiang MX Hong X Liao BB Shi SZ Lai XF Zheng HY. Expression profiling of TRIM protein family in THP1-derived macrophages following TLR stimulation. Sci Rep (2017) 7:42781. doi: 10.1038/srep42781 28211536 PMC5314404

[56] Xiao T Wan J Qu H Li Y. Tripartite-motif protein 21 knockdown extenuates LPS-triggered neurotoxicity by inhibiting microglial M1 polarization *via* suppressing NF-kappaB-mediated NLRP3 inflammasome activation. Arch Biochem Biophys (2021) 706:108918. doi: 10.1016/j.abb.2021.108918 33992596

[57] Lu J Xie L Liu C Zhang Q Sun S. PTEN/PI3k/AKT regulates macrophage polarization in emphysematous mice. Scand J Immunol (2017) 85:395–405. doi: 10.1111/sji.12545 28273403

[58] Vergadi E Ieronymaki E Lyroni K Vaporidi K Tsatsanis C. Akt signaling pathway in macrophage activation and M1/M2 polarization. J Immunol (2017) 198:1006–14. doi: 10.4049/jimmunol.1601515 28115590

[59] Zhou D Huang C Lin Z Zhan S Kong L Fang C. Macrophage polarization and function with emphasis on the evolving roles of coordinated regulation of cellular signaling pathways. Cell Signal (2014) 26:192–7. doi: 10.1016/j.cellsig.2013.11.004 24219909

[60] Luyendyk JP Schabbauer GA Tencati M Holscher T Pawlinski R Mackman N. Genetic analysis of the role of the PI3K-akt pathway in lipopolysaccharide-induced cytokine and tissue factor gene expression in monocytes/macrophages. J Immunol (2008) 180:4218–26. doi: 10.4049/jimmunol.180.6.4218 PMC2834303 18322234

[61] Lee JH Liu R Li J Zhang C Wang Y Cai Q. Stabilization of phosphofructokinase 1 platelet isoform by AKT promotes tumorigenesis. Nat Commun (2017) 8:949. doi: 10.1038/s41467-017-00906-9 29038421 PMC5643558

[62] Wang G Li X Wang H Wang Y Zhang L Zhang Le. Later phase cardioprotection of ischemic post-conditioning against ischemia/reperfusion injury depends on iNOS and PI3K-akt pathway. Am J Trans Res (2015) 7:2603–11. PMC4731660 26885260

[63] Choi W-S Seo Y-B Shin P-G Kim W-Y Lee SY Choi Y-J. Veratric acid inhibits iNOS expression through the regulation of PI3K activation and histone acetylation in LPS-stimulated RAW264.7 cells. Int J Mol Med (2015) 35:202–10. doi: 10.3892/ijmm.2014.1982 25352364

