# Supplementary figures and images for: TRIM21 aggravates cardiac injury after myocardial infarction by promoting M1 macrophage polarization
Source: Front Immunol. 2022 Nov 10;13:1053171. doi: 10.3389/fimmu.2022.1053171 (PMC9684192; doi:10.3389/fimmu.2022.1053171)

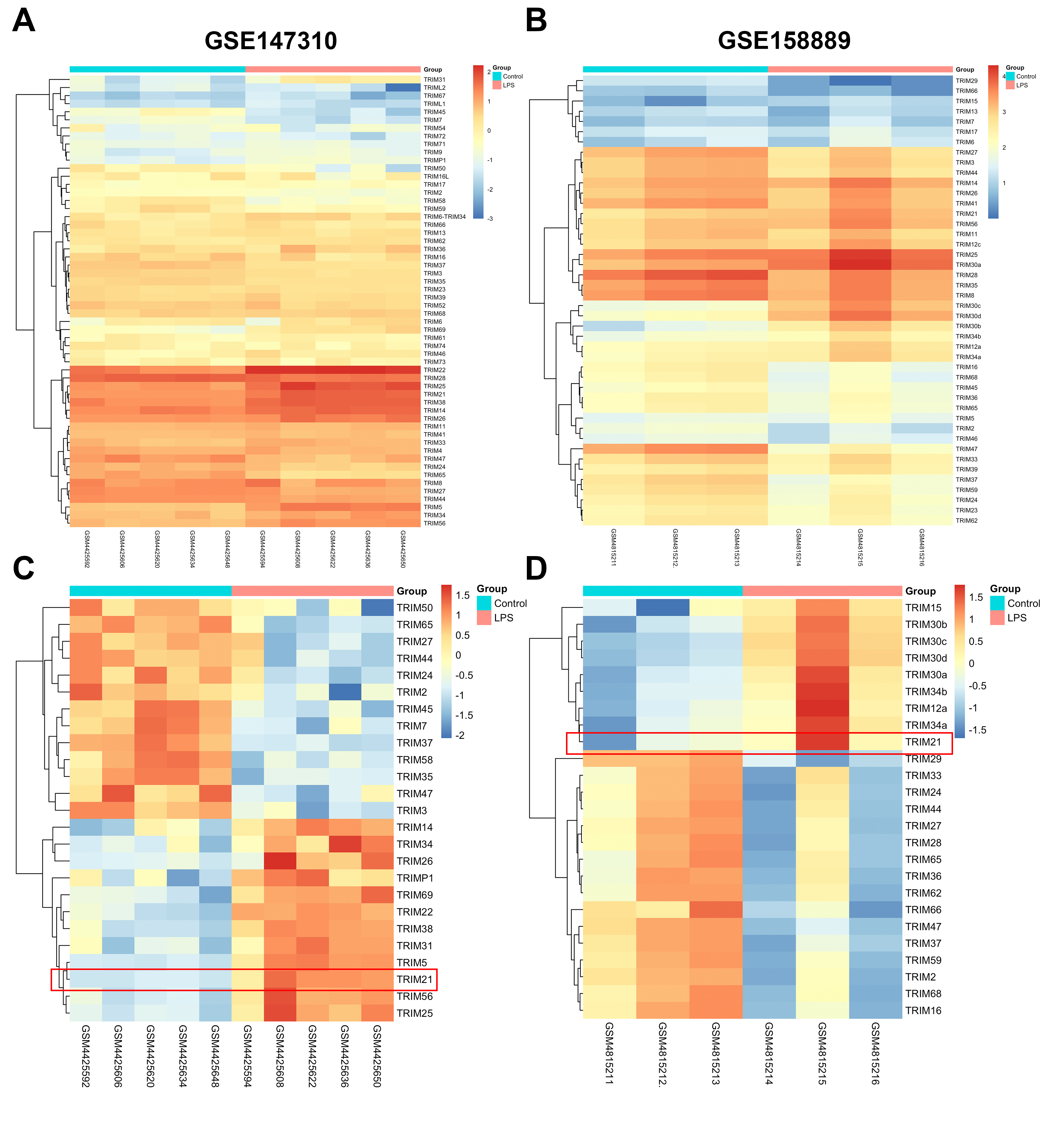

Supplement: Supplementary Figure 1 — Differentially expressed of TRIMs family in LPS-stimulated macrophages. Heatmap shows the log10 (normalized counts +0.01) of TRIMs family from (A) GSE147310 and (B) GSE158889 microarray. Top 25 differentially expressed genes (DEG) in (C) GSE147310 and (D) GSE158889 microarray. Red represents high expression, blue means low expression. [file Image_1.jpeg]

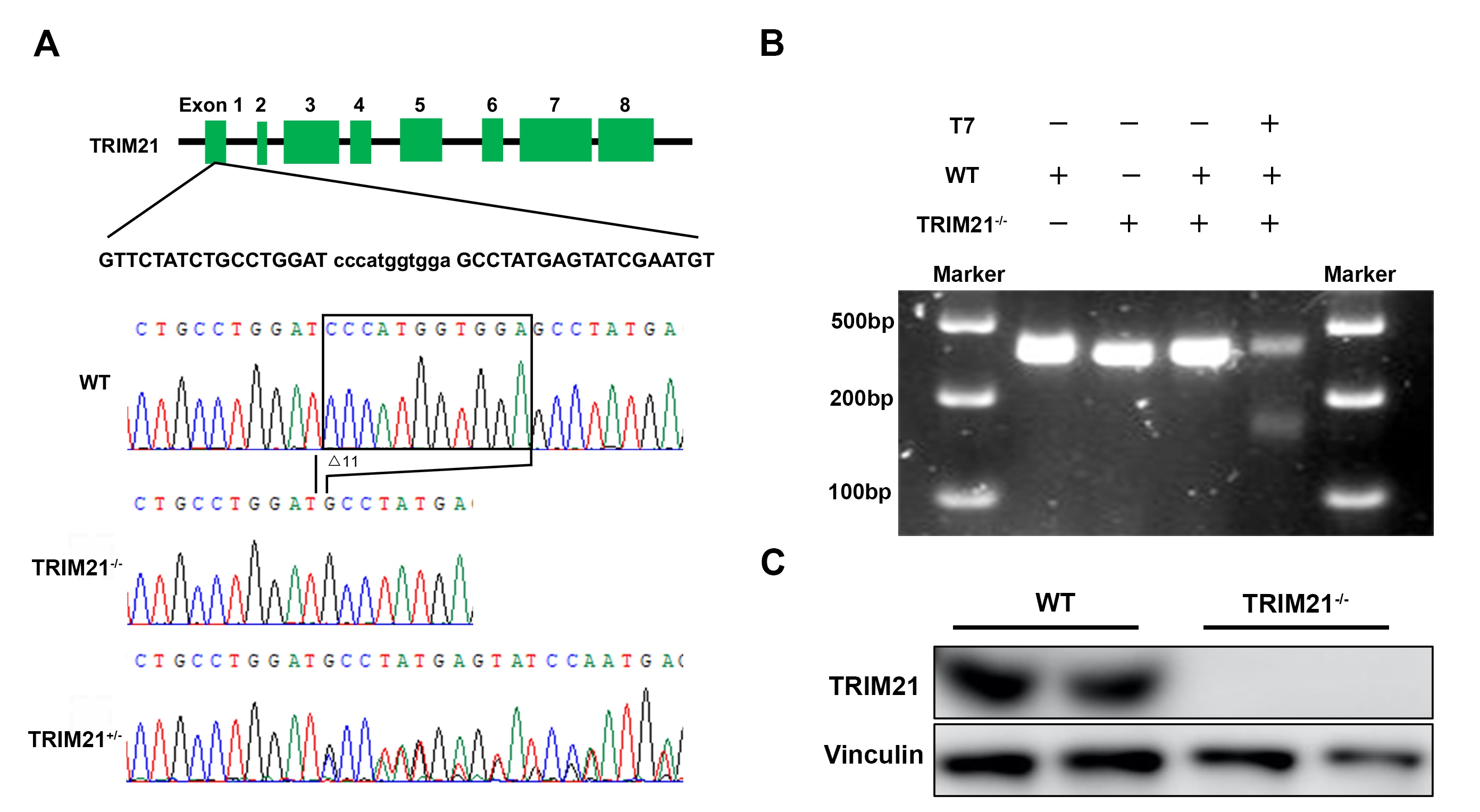

Supplement: Supplementary Figure 2 — Establishment and verification of TRIM21 knockout in mice. (A) Diagram of the partial TRIM21 gene body showing the TALEN (Transcription Activator-Like Effector Nuclease) target sites (top), and DNA sequences of the cloned polymerase chain reaction products in WT, TRIM21-/- and TRIM21 heterozygous (TRM21+/-) mice (bottom). (B) Agarose gel images of digestion products from WT and TRIM21KO mouse tail digested with a T7E1 assay. (C) Western blot assay shows the level of the TRIM21 protein in the WT and TRIM21-/- hearts. Data are representative of 3 independent experiments. n = 2 mice/group. [file Image_2.jpeg]
