# Supplementary material for: TRIM21 aggravates cardiac injury after myocardial infarction by promoting M1 macrophage polarization
Source: Front Immunol. 2022 Nov 10;13:1053171. doi: 10.3389/fimmu.2022.1053171 (PMC9684192; doi:10.3389/fimmu.2022.1053171)
Supplement: Supplementary file 3 [file Table_1.docx]

| Supplement Table 1 Sequences used in qRT-PCR | | |
| --- | --- | --- |
| Primer | Forward Sequences (5′-3′) | Reverse Sequences (5′-3′) |
| TRIM21 | GGCGTTGTCTCCTTCTAC | AGTGAGCAGCAGTATCCA |
| iNOS | ACTCAGCCAAGCCCTCACCTAC | TCCAATCTCTGCCTATCCGTCTCG |
| TNF-α | CCCTCACACTCAGATCATCTTCT | GCTACGACGTGGGCTACAG |
| IL-6 | GGCGGATCGGATGTTGTGAT | GGACCCCAGACAATCGGTTG |
| Arg1 | CTCCAAGCCAAAGTCCTTAGAG | AGGAGCTGTCATTAGGGACATC |
| Ym-1 | AGAAGGGAGTTTCAAACCTGGT | CTCTTGCTGATGTGTGTAAGTGA |
| IL-10 | GCTCTTACTGACTGGCATGAG | CGCAGCTCTAGGAGCATGTG |
| GAPDH | GTGTTCCTACCCCCAATGTG | CATCGAAGGTGGAAGAGTGG |
